# A feedback mechanism controls rDNA copy number evolution in yeast independently of natural selection

**DOI:** 10.1371/journal.pone.0272878

**Published:** 2022-09-01

**Authors:** Vicente Arnau, Marina Barba-Aliaga, Gaurav Singh, Javier Ferri, José García-Martínez, José E. Pérez-Ortín

**Affiliations:** 1 Institute of Integrative Systems Biology (I2SysBio), University of València and Consejo Superior de Investigaciones Científicas (CSIC), València, Spain; 2 Instituto de Biotecnología y Biomedicina (Biotecmed), Universitat de València, Burjassot, Spain; Tulane University Health Sciences Center, UNITED STATES

## Abstract

Ribosomal DNA (rDNA) is the genetic loci that encodes rRNA in eukaryotes. It is typically arranged as tandem repeats that vary in copy number within the same species. We have recently shown that rDNA repeats copy number in the yeast *Saccharomyces cerevisiae* is controlled by cell volume via a feedback circuit that senses cell volume by means of the concentration of the free upstream activator factor (UAF). The UAF strongly binds the rDNA gene promoter, but is also able to repress *SIR2* deacetylase gene transcription that, in turn, represses rDNA amplification. In this way, the cells with a smaller DNA copy number than what is optimal evolve to increase that copy number until they reach a number that sequestrates free UAF and provokes *SIR2* derepression that, in turn, blocks rDNA amplification. Here we propose a mathematical model to show that this evolutionary process can amplify rDNA repeats independently of the selective advantage of yeast cells having bigger or smaller rDNA copy numbers. We test several variants of this process and show that it can explain the observed experimental results independently of natural selection. These results predict that an autoregulated feedback circuit may, in some instances, drive to non Darwinian deterministic evolution for a limited time period.

## Introduction

The eukaryotic genome is composed of both unique and repetitive DNA sequences. Amplifications and deletions of repeats are generators of genomic changes that potentially help organisms to better adapt to a variable environment [1]. It has been shown that the number of tandem repeats is prone to vary more easily than any other genome feature in both lower and higher eukaryotes [2–4]. When the repeated sequence contains a gene, copy number variation can provide a plethora of different daughter cells with variable expression levels, which is useful for coping with new environments. This is the case of the *CUP1* and 35S rRNA genes in the yeast *Saccharomyces cerevisiae*, which can be amplified or reduced in copy number to adapt to either copper exposure (*CUP1*) or new growth rate conditions (*rDNA*) [5,6]. Similar examples have been observed in cancer cells treated with epidermal growth factor receptor (*EGFR*) tyrosine kinase inhibitors [7], in *Leishmania* parasites [8], and also in mouse fibroblasts exposed to high methotrexate concentrations [9]. In all these cases, there is a marked tendency to amplify repeated loci, which can be maintained as tandem repeats in the chromosome [10] or be extruded as extrachromosomal circles [5,7].

**Fig 1:**
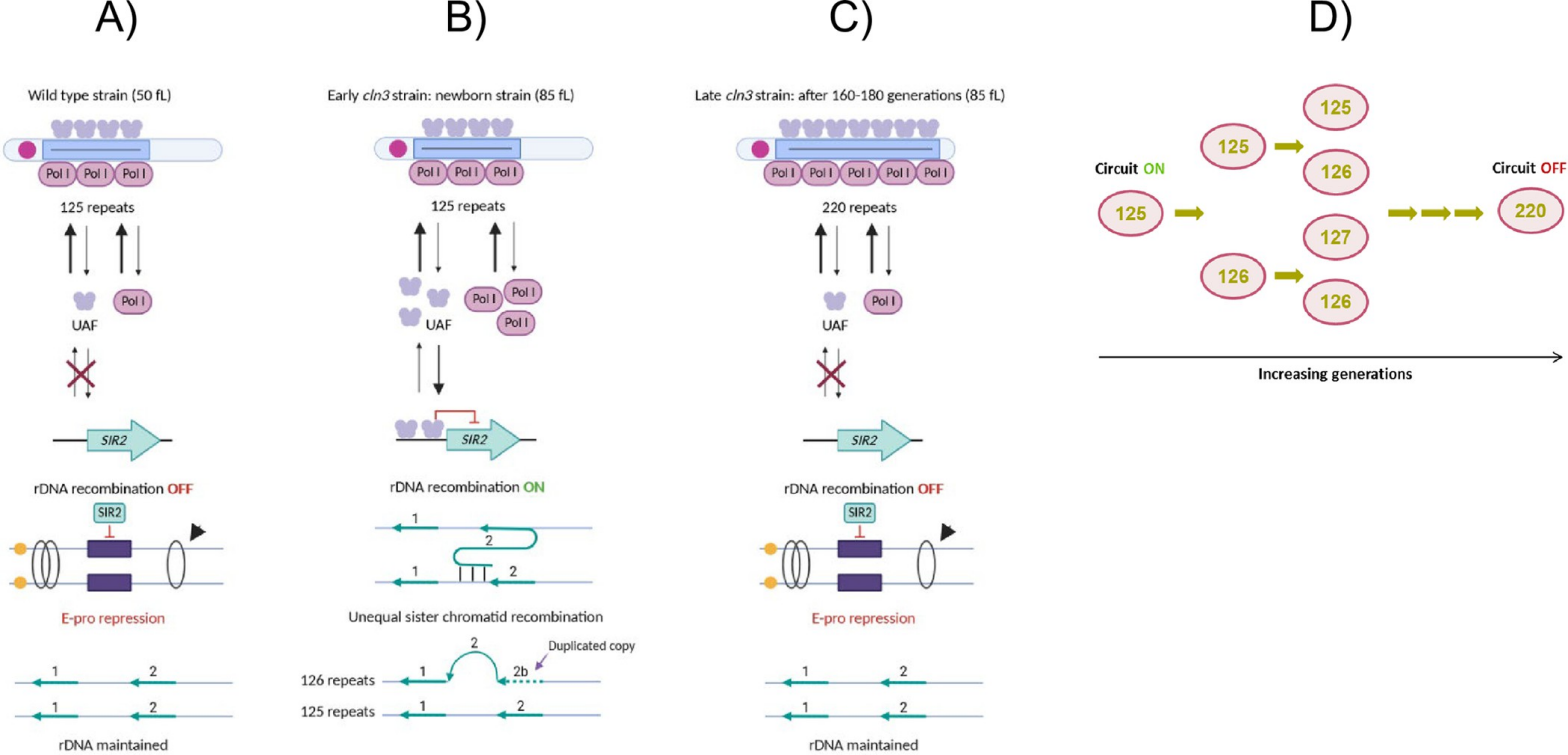
Model for the regulation of the rDNA copy number in the yeast *S*. *cerevisiae* by cell volume using the “musical chair” feedback circuit. A,B) In a newly made *cln3* strain, the cell volume is 85 fL, which is much bigger than its parental wild type (50 fL). The number of rDNA repeats is, however, the same: 125. As the cellular concentration of most proteins is identical in all cells [21], the number of UAF and RNA pol I molecules should increase proportionally to cell volume and in such a way that in the smaller wild type, the number of UAF molecules is in excess of the rDNA repeats, which leaves many free nucleoplasmic UAF molecules. The UAF also possesses repressor activity for the *SIR2* gene promoter which then becomes repressed. The low Sir2 histone deacetylase activity avoids the repression of the recombination in the rDNA locus (see references 16,17 for a more detailed description). The unequal sister chromatid recombination (USCR) works only in a way that it amplifies copies (1 or more) in one of the daughter chromatids [14]. C) When the amplification of the rDNA repeat copy number reaches a number (about 220–250 copies) that sequesters most free nucleoplasmic UAF molecules, the *SIR2* promoter becomes derepressed and Sir2 activity blocks USCR to stop rDNA repeat amplification. The rDNA repeat number is then maintained with generations as in a wild type strain. D) The number of repeats grows stepwise from 125 to about 220 copies, on average, in a yeast culture for about 160–180 generations in a heterogeneous population of yeast *cln3* cells until the feedback circuit is off.

rDNA in eukaryotes is an evolutionary conserved cluster of tandemly arrayed repeating units. Each repeat contains the gene for the 35-45S rRNA precursor and other genetic elements [11,12]. The number of repeats varies between species and also within the same species, and can be related to cells’ proliferative capacity. Its variation may contribute to the disease risk in several cancers and mental disorders [13]. In *S*. *cerevisiae*, the rDNA cluster varies between 40 and 350 copies, and is very dynamically rearranged [14,15]. In a previous study, we found that the rDNA repeat number evolves from 125 to about 220–250 copies along 160–180 generations in a freshly made (“early”) *cln3* yeast mutant [16]. *CLN3* is a gene that encodes a G1 cyclin involved in cell cycle progression. It activates G1 to S phase transition [17]. Its mutation causes yeast cells to remain in G1 for longer time, increasing their cell size. In fact, *cln3* has a much larger (roughly double) cell volume than the wild type. However, the actual number of rDNA repeats in an early mutant is the same as in the parental wild type and is, therefore, smaller than that needed to maintain the rRNA synthesis rate for such a bigger volume. We demonstrated that the mechanism that acts during such evolution follows the “musical chair model” proposed by T. Kobayashi [18]. This model states that unequal sister chromatid recombination (USCR) in *S*. *cerevisiae* is active only when *SIR2* gene expression is repressed by free molecules of the upstream activator factor (UAF). The free UAF concentration depends inversely on the ratio between the number of rDNA repeats and the cell volume because the UAF binds with high affinity to the rDNA promoter. The free UAF concentration is negligible for a wild type with 125 repeats, but is high for an early *cln3* mutant with 125 repeats, and almost double cell volume [16,18]. This is because the total (free + bound) UAF concentration is constant (like most cellular proteins) with increased cell volume, but the number of rDNA targets is not immediately increased in early *cln3* cells. Activation of USCR by free UAF molecules triggers the possibility of increasing the repeat copy number. This acts as a feedback circuit that maintains USCR active while the repeat copy number remains under 220–250 copies (Fig 1). By assuming natural selection, we predicted that the evolved (“late”, 220 repeats) *cln3* mutant better fits rRNA synthesis and should grow more quickly than the early (125 repeats) one. However, here we find that this is not the case, but quite the opposite in fact. Indeed, previous researchers have also found that other yeast strains with a smaller rDNA repeat number that evolve to a bigger one do not show a slower growth rate than those with more rDNA repeats [19,20].

We hypothesize that as USCR in the yeast *S*. *cerevisiae* only works in the rDNA amplification sense [14], the observed evolution could be driven by a non Darwinian process based on a feedback circuit that causes deterministic directional evolution. Using a cellular automaton model [22–24], we demonstrate that such a circuit can work independently of natural selection while the circuit is on. The prediction of this model very well matches published results on rDNA amplification in yeast [16,19,20].

## Materials and methods

### Yeast strains, media and growth conditions

All the *S*. *cerevisiae* strains herein used are listed in S1 Table. Yeast cells were grown in liquid YPD (2% glucose, 2% peptone, 1% yeast extract). The *cln3Δ* mutants (“early”) were obtained by gene disruption and employed in an evolution experiment as described in [16]. Briefly, yeast cells were grown in 20 mL cultures at a starting cell density of 10000 cells/mL for 24-hour periods and re-inoculated every 11–12 generations for 25 days (250–280 generations). The experiment was done in quadruplicate.

The apparent growth rate (GR app) of the wild-type and *cln3* mutants was calculated by growing 50 mL of yeast cultures in 250 mL flasks with shaking (190 rpm) at 30°C. Aliquots were taken every 60 min in the exponential phase and their OD_600_ were measured. GRs (h^-1^) were calculated from the growth curves in YPD for three independent colonies of the early and old *cln3* mutants. The experiment was repeated twice using the wild-type and Euroscarf *cln3* controls. Therefore, the given data in Table 1 come from six repeats. Generation times (in hours) were calculated as ln2/GR.

**Table 1:** Yeast strains’ growth features.

**Strain**	**GR (app, h** ^**-1**^ **)**	**% dead cells (F)**	**GR (actual, h** ^**-1**^ **)**
BY4741	0.46± 0.01	2.6± 0.27	0.45
Early *cln3*	0.45± 0.03	4.4± 0.52	0.43
Late *cln3*	0.37± 0.05	2.2± 0.48	0.36
Euroscarf *cln3*	0.37± 0.06	1.4± 0.13	0.37

GR (actual) = GR (apparent)–DR *GR*: *growth rate*.

DR = F x GR (apparent) *DR*: *death rate*.

F: fraction of dead cells.

F = dead cells/total cells.

GR (app) = total cells/time.

DR = dead cells/time.

### Cell volume and viability measurements

The median values of the population’s cell volumes were obtained with a Coulter-Counter Z series device (Beckman Coulter, USA), as previously described [25]. The absolute values (in fL) are shown in S1 Table. The percentage of dead cells (F) was determined by a flow cytometry analysis of propidium iodine-stained cells [26] from five repeats of the experiment. The actual growth rate (GR actual) was calculated using the equations presented in Table 1.

### Evolutionary simulations

We used cellular automaton models [22–24]. The first model (model A, S1 Fig) is made up of N states, with as many as the possible number of gene copies that a cell has. If we have cells with from 125 to 220 rDNA copies, we have N = 95 different states. The number of cells in each state changes over time. We start from an initial situation in which all the states have 0 cells, except for state 125, which has 10000 cells. In each cell generation, two new cells are generated: one with the same copy number and the other with an integer increase in copy number, which we call “Delta”. For the non integer Delta (e.g. Delta = 1.3), this means that 70% of the 10000 dividing cells have an increase of one copy and 30% have an increase of two copies. One feature of our model is that when cells reach their maximum rDNA copy number, which is 220 copies in our case, they divide into two cells with the same copy number as the parent cell because recombination ceases as predicted by the “musical chair model” [18]. We can simulate continuous cell division until system saturation occurs. We assume saturation when 98% cells have 220 copies (model A1). Model A1 is binary insofar as it considers that the Sir2 repression of rDNA recombination is absent until the yeast strain reaches 220 copies. The actual situation probably involves Sir2 activity increasing continuously from the lowest level at 125 copies to the highest level at 220 copies. We implemented an alternative non binary model (A2), in which a linear increase in Sir2 activity (decrease in rDNA recombination) occurs (S2 Fig). As expected this model makes evolution slower for a given Delta value because the percentage of cells undergoing recombination lowers with the number of repeats. This causes the average copy number increase per generation to not be constant (as in Model A1), but to continuously lower. The Model A2 previously described was also derived from Model A3, that is, from a reseeding culture. The results obtained were mostly identical to those shown in S2 Fig. We also developed an alternative model (Model A3) by reseeding 10000 cells every 12 generations (which was done in the evolution experiment described in [16]). To continue applying the model, these 10000 cells are randomly chosen from the set of cells in the system after 12 generations. In Models A1/A2/A3, cells divide every 97 minutes (5820 seconds), which is the generation time observed for the early *cln3* cells (Table 1). See the S1 Appendix for a description of the pseudocode. Implementation details, as well as two source codes, can be downloaded from https://www.uv.es/varnau/modelo/MODEL_A1.c and https://wwwuv.es/varnau/modelo/MODEL_A2.c.

In Model B, we follow the same division strategy as in Model A3, but in this case, the time that it takes for one cell to divide depends on its number of copies. We use a generation time increase (GTI) factor, which means that for every additional rDNA copy of the cell, it takes more or less time to divide. We assume that there can be a maximum variation of ±15% in the growth rate between the different states of the *cln3* cells (from the *early* and *late* data in Table 1). This represents ±855 s. We also assume that GTI is constant during the increase in the rDNA copy number. Hence for a total increase of 95 copies, each rDNA copy represents about 9 s. GTI can take positive or negative values. S3 Fig shows the scheme when GTI = -9 s, where the cells with 125 copies take 5820 s to divide, and with divisions 9 s earlier for each extra copy that a cell has. When a cell reaches 220 copies, it divides into two cells of 220 copies. A similar scheme, but with a positive GTI, can be applied. Both implementation details and source codes can be downloaded from https://www.uv.es/varnau/modelo/MODEL_B.c.

## Results

### Newly made *cln3* mutants have genomic and physiological characteristics that differ from those of *cln3* strains in reference collections

We transformed a BY4741 wild-type strain with the KanMX4 cassette to obtain a *cln3Δ* mutant following standard protocols [27]. The newly created *cln3Δ* strain (“early”) has the same cell volume (85 fL) as a *cln3Δ* strain from a Euroscarf collection, but only 125 rDNA repeats (ref. 16; see Fig 1). The same results were obtained with the early *cln3Δ* strains obtained after the sporulation of a heterozygote *CLN3/cln3* diploid (not shown). An evolutionary experiment showed that the rDNA copy number gradually increased to 220–250 copies in about 160–180 generations (“late strain”; ref. 16) that also has a cell volume of 85 fL (S1 Table).

Unexpectedly the growth rate of the early *cln3Δ* was slightly faster than that of the evolved late *cln3Δ* and the Euroscarf collection *cln3Δ* (late) strains (Table 1). This result predicts that early cells cannot be substituted for the evolved ones simply by growth rate competition. We wondered if the mortality rate of early and late strains could be differential and affect the predicted results. We found that early cells had a slightly higher percentage of dead cells than the late and wild-type ones (Table 1). However, the calculation of the actual growth rate by considering correction by cell mortality still predicted an evolutionary advantage for early cells over late ones (Table 1).

### Modeling the increase in rDNA copy number suggests a deterministic evolution mode

We previously proposed that an increase in rDNA copy number in an early *cln3* strain is based on a mechanism proposed by T. Kobayashi called the “musical chair model” [16,18,28]. This mechanism is a feedback circuit (Fig 1A–1C) that is activated by the low rDNA concentration which occurs in a 125-repeat strain with a large cell volume (early *cln3*). The mechanism for copy number changes in yeast rDNA described in the literature is unequal sister chromatid recombination (USCR) [2,11,14,29]. Interestingly, although the original proposals for USCR in yeast state that one sister chromatid increases and the other decreases in repeat number [2,11,29,30] because of unequal crossing over [11,31], the USCR mechanism proposed by T. Kobayashi [14] for rDNA amplification in small-repeat-copy-number strains produces one daughter genome with an increased copy number, whereas the other one remains with same copy number as the mother cell (Fig 1B).

**Fig 2:**
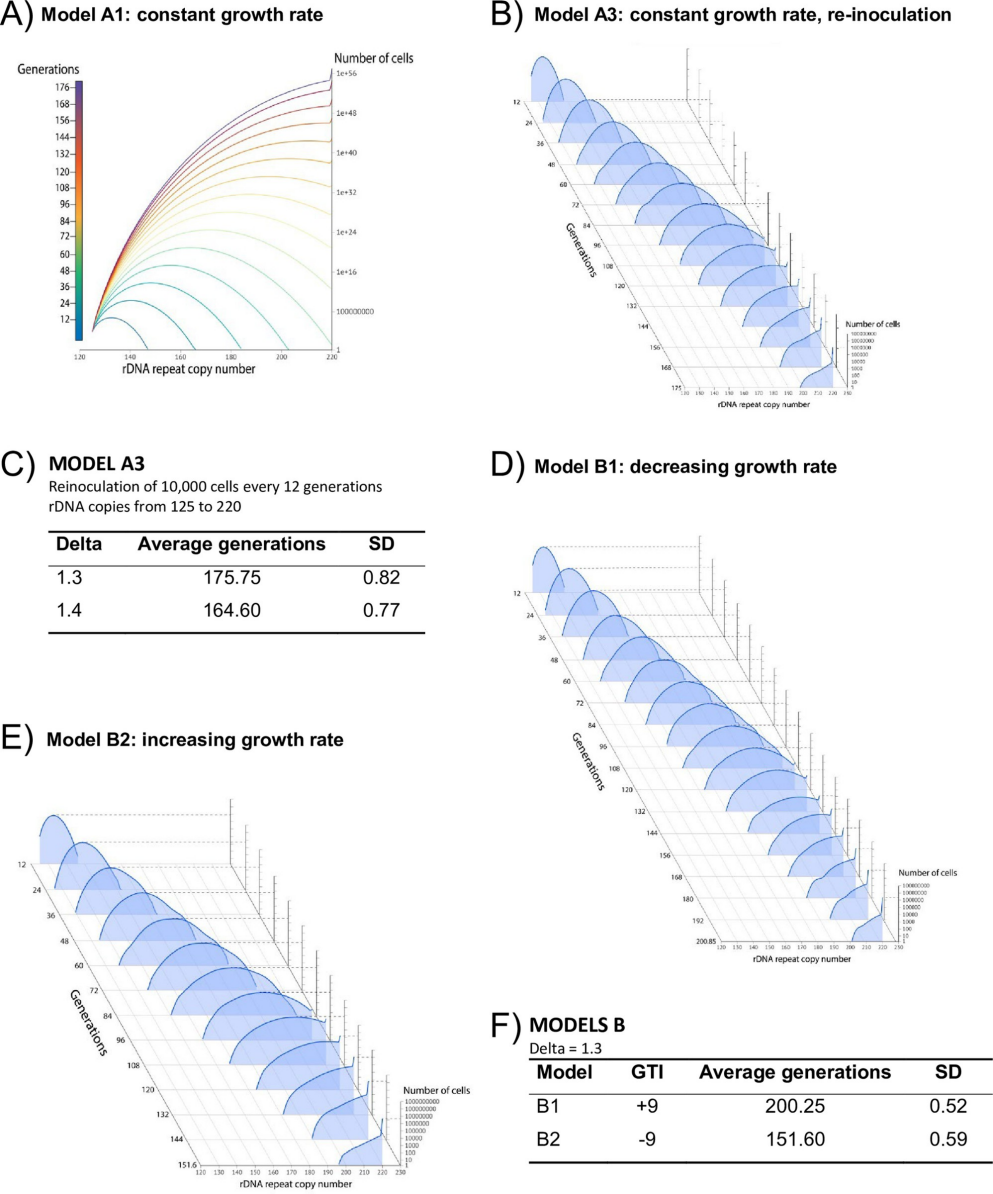
Cellular automaton model accurately predicts the experimentally observed evolution of early *cln3*. A) Model A1 shows the predicted evolution during the generations of the cell population according to the scheme shown in S1 Fig. Population curves are shown only for multiples of 12 generations up to the generation where >98% of cells have 220 repeats. B) Model A3 shows the predicted evolution for the re-inoculation of 10000 cells every 12 generations. This model has a stochastic parameter that produces variation during each program run. C) A summary of the results obtained with Model A3 after 10 runs. Average and standard deviations (SD) are shown for the increased parameter per generation (Delta) of 1.3 and 1.4. D, E) Models B show the influence when considering that cells have slightly different growth rates (or generation times) depending on the repeat number. B1 shows the cells with a larger copy number with slower growth rates (9 s less generation time per copy). Model B2 shows the cells with a bigger copy number with faster growth rates (9 s more generation time per copy). F) Summary of the results obtained with models B after 10 runs. Average and standard deviations (SD) of the number of generations are shown for an increased or decreased generation time increase (GTI) per rDNA copy increase using a Delta of 1.3. See S3 Fig for a scheme of the algorithm.

Therefore, we decided to model the evolution of a cell (early *cln3*) in which the musical chair circuit is activated by considering that the sister cells in every genome duplication can either increase or maintain the rDNA copy number, but cannot decrease the repeat number or, alternatively, this occurs in a very low proportion. We used a cellular automaton model [22–24] in which we defined the original rDNA copy number as 125 repeats and the final copy number as 220 repeats. The starting number (10000 cells/mL) was used as inoculum according to our previously published data [16]. The model (Model A1, in Fig 2A and S1A Fig) predicts deterministic evolution, during which the population’s average copy number increases because of the continuous increase in copy number in the daughter cells after each replication without a compensatory decrease in the other one (Fig 1B). The model stops increasing rDNA repeats when the final number (220) is reached. After about 175 generations, we calculate that more than 98% cells have 220 repeats when the increase per replication is 1.3 repeats on average (“Delta” in Fig 2A and S1B Fig). This copy number increase per generation falls within the range experimentally observed by other authors [19,20]. We made some modifications to this simplest model. We first analyzed the effect of considering that the decrease in *SIR2* repression by UAF is not binary (on→off), but decreases continuously from 100% at 125 repeats to 0% at 220 repeats. In this model, the Delta factor should increase to 11 repeats (S2 Fig) to fit the experimentally observed number of generations (160–180). We also performed a model by considering the re-inoculation of 10000 cells/mL every 12 generations (as in our original experiment: ref. 16) given the potential bottleneck effect. Our results show that the bottleneck effect is very mild because it delays by less than 1 the number of generations required to obtain the final 220 repeat number (Model A3, Fig 2B and 2C). Then we analyzed the effect of limiting the number of replications that a mother yeast cell can make because the aging of the mother limits the number of allowed generations [15,32]. We found that no changes in predictions happen when this feature is taken into account (not shown). With all these virtual experiments, we conclude that our simple deterministic model quite well explains the actual results that we previously obtained [16].

The previous model does not take into account the difference in the growth rate observed between early and late *cln3* strains (Table 1). Other authors [19,20] have observed that yeast strains with a small rDNA repeat copy number do not display significant growth rate differences, but provide no quantitative data. As we found that the early *cln3* strain has an approximately 15% faster growth rate than the late one (Table 1), we wished to discover how a difference in the growth rate between early and late *cln3* strains could affect the results. We modified our cellular automaton model (Model B) to take into account differences in the growth rate between the cells with distinct rDNA repeats. We implemented the model for the case of a 15% faster growth rate (lower generation time) and also for a hypothetical 15% decrease. Our model B assumes that the increase or decrease in the growth rate is gradual and, because of that, every single added repeat has an effect of ±9 s (generation time increase per copy: GTI) for a total increase of 855 s in a 95 copy increase (125→220) in rDNA repeats. Model B predicts that the decrease/increase in the growth rate impacts the number of generations required to obtain the final 220 repeats by about ±12–13%, which is slightly below the percentage of growth rate variation (Fig 2D–2F). Growth rate changes >15% would have a stronger impact, but have never been observed for yeast strains with variable rDNA copy number. So we decided to apply our model to the results of the rDNA amplification reported by other authors in different strains and experimental situations in yeast [19,20]. We found that our model can explain those results when using some reasonable alternatives for the average increase repeat (Delta) and the GTI (see Table 2).

**Table 2:** Several alternative possibilities using the Model B1 parameters to fit published experimental data.

	Published results			Modeling		
	Initial copies	Final copies	Observed generations	GTI*	Delta**	Predicted generations
				0	2.3	80
Kobayashi et al.	80	150	80	-6	2.0	82.7
1998 [19]				-9	2.0	80.6
				0	2.05	60
Jack et al. 2015 [20]	35	80	60	-9	1.90	59.7
				-9	1.80	63.5

*GTI in seconds

**Delta is the average copy number increase per generation.

We also consider the possibility of USCR giving two daughter cells, one with amplification and the other one with symmetrical reduction in rDNA copy numbers. In this case, if growth rates are constant or decrease with an increase in copy number, as experimentally found [16,19,20], the system does not evolve to amplification (125→220), but evolves to a widening the copy number range in the case of a constant growth rate or to a reduction in average copy number, in the case of growth rate decrease in the late *cln3* strain (not shown).

Thus we propose that yeast cells activate the Sir2/UAF circuit called “musical chair” because the sudden volume increase caused by *CLN3* deletion provokes evolution toward an rDNA increase irrespectively of classic natural selection forces until the circuit is turned off. This is because the amplified number of repeats sequesters most of the UAF molecules in the cell that are now unable to repress *SIR2* transcription (see Fig 1). This process may occur, albeit at a different speed, even in those cases in which evolving strains have slightly different experimentally observed growth rates (Fig 2D–2F). This modeling proposes that natural selection can speed up or delay the observed evolution, but cannot prevent it.

## Discussion

In a previous paper, we showed that a fresh yeast mutant (early *cln3*) with an increased cell volume evolves to a new strain (late *cln3*) with a different genome composition (more tandem rDNA repeats). The present study into the evolution process shows that this is merely because a feedback circuit exists and is activated by the sudden increase in cell volume, which occurs during the first generation after *CLN3* deletion.

rDNA locus evolution takes place in the mitotic cycle only during DNA replication, and does so with a limited copy number increase per generation [14]. rDNA evolution to a large number of rDNA repeats in yeast strains entails a deficit in rDNA copies and happens in relatively few generations, as previously shown by other laboratories [19,20,33]. It has been predicted to be caused by the selection of the fastest-growing cells [31]. However, more recent results [16,19,20] show that strains with a small copy number do not have slower growth rates than their bigger-copy-number counterparts. The present study proposes a model in which the increase in rDNA repeats is because yeast USCR works only in the increasing copy number sense [14,19]. Our modeling reveals that such a circuit provokes an average population increase in rDNA repeats in a number of generations that is compatible with both the experimental observations of average increase per generation [19,20] and the observed number of 160–180 generations [16,19,20]. Our model works without selection. In it mutation would be the only evolutionary force, but would also work within the growth rate increase range (<15%) observed for the *cln3* mutants in relation to the wild type.

**Fig 3:**
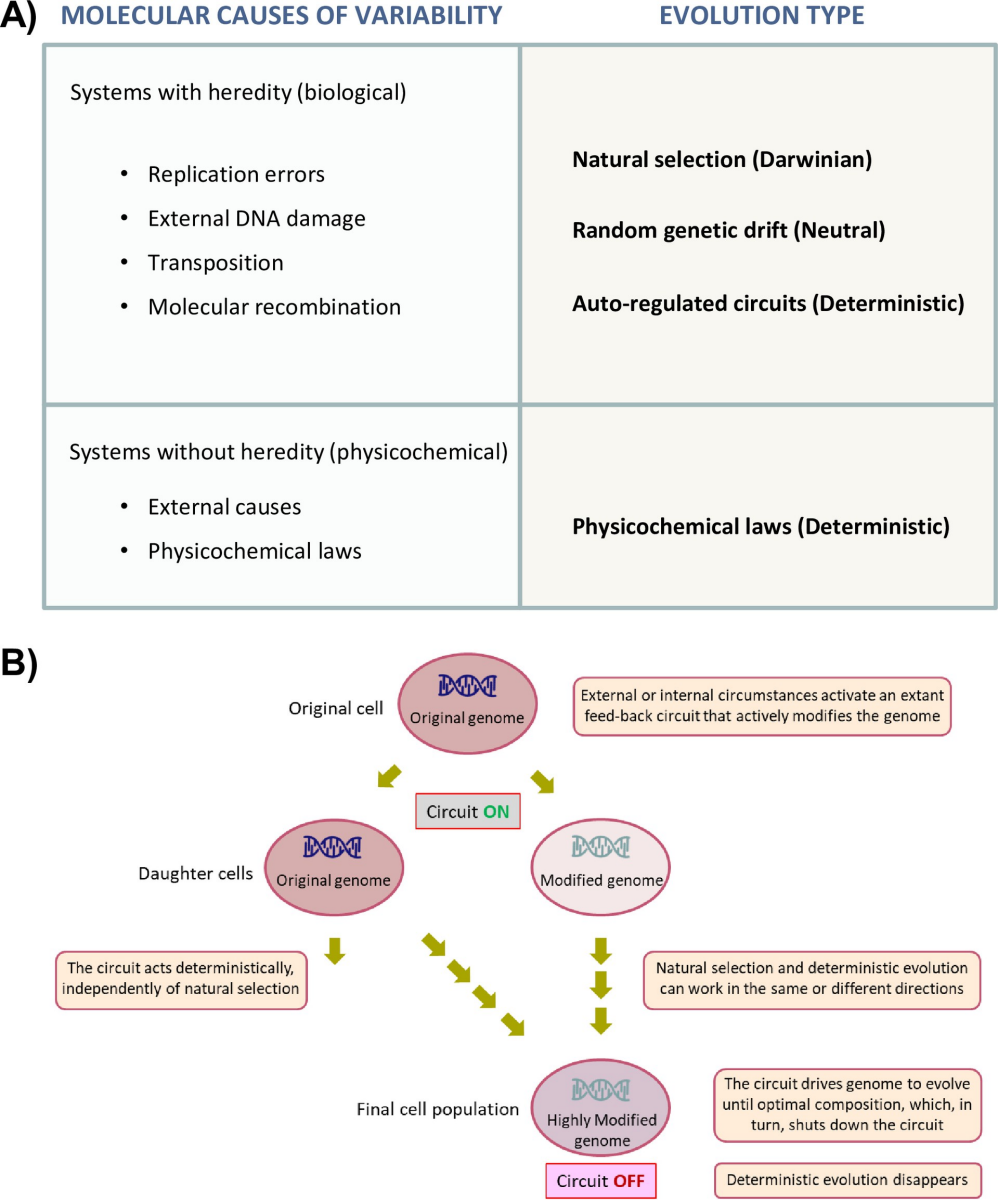
Deterministic evolution features in living systems. A) Evolving systems need to develop a molecular source of variability and a downstream mechanism that determines the type of evolution. Deterministic evolution occurs in other non biological systems, follows physical or chemical laws in the absence of heredity, and independently of the selective pressures that conduct most biological evolution. B) Some extant feedback circuits developed for any specific purpose can be triggered by internal or external changes, and in such a way that it leads to deterministic evolution.

The evolution of tandem arrays has been previously modeled by other authors [10]. In their study, repeat number amplification occurs because the equilibrium between the removal of repeats and their amplification is displaced toward amplification due to the continuous action of an undetermined amplification mechanism. Such a model was conceived for a kind of tandem repeats that, unlike the rDNA ones, are not under obvious selection because they confer no selectable phenotype (e.g. minisatellites; ref. 4). Here we extend this idea to the rDNA repeats that play a very important role in cell physiology. Thus we hypothesize that autoregulated circuits can drive yeast cells to deterministically evolve (Fig 3A) to a final new steady state for their genome composition (Fig 3B). This is because there is a source of genetic variation (e.g. recombination) capable of providing a unidirectional drift while the circuit is on, but the actual growth rate (that conditions the progeny number) of evolved cells does not deviate very much from the original one. This evolution occurs at the same time as that conducted by natural selection, which can be added to it to speed up or slow down the final result (Fig 2D–2F).

We suggest that the rDNA amplification by cell volume increase that we saw in the *cln3* mutant represents a new type of evolutionary process that differs from Darwinian and neutral evolution (Fig 3A) by acting during limited periods in some specific cases. It is not partially deterministic and partially contingent as natural selection can be [34]. Nor is it a case of genetic canalization [35] because, in those cases, natural selection is the only evolutionary force that acts upon restricted variability. Our new model of evolution is strongly deterministic insofar as it works under strong architectural constraints because variability occurs only in a predetermined way whenever the circuit is active. Such a process is like the physico-chemical evolution of nonliving systems [36] and co-exists in *cln3* yeasts with Darwinian and neutral evolution (Fig 3A).

We are able to consider what the features of any deterministic circuit can be to act as an evolutionary force in a living system. The first essential feature of a deterministic circuit would be the capacity to be induced by an external or internal effector (Fig 3B). Genome changes should be caused by a molecular mechanism, such as recombination. The most straightforward mechanism for this appears to be alterations in gene copy number. In fact, variation in gene copy number has been shown to be a frequent cause of evolution in different species for both the RNA pol I gene (rDNA) and the RNA pol II genes in several eukaryotes, such as yeasts [5,6,37], cancer cells [7,38], *Leishmania* [8,39] or mouse fibroblasts [9]. It may occur by either tandem repeat amplification/contraction or the generation of extrachromosomal DNA (ecDNA). Amplification may happen in a few cell replications [19,20] and be induced by an environmental agent [7,9]. However, all the cases reported to date argue that the evolution of cells always happens by natural selection because the cells with a higher expression of the (amplified) gene are better adapted to the new environment. In fact, it has been shown that the mechanism of variability generation may be replication and/or transcription [19], but this mechanism does not act *per se* in evolution toward copy number amplification [6].

A second feature of a deterministic circuit would be that it should act as continuously constrained in one direction. Mutational force should be so strong and directional that it can overcome selection and reduce an evolving population’s fitness. As previously mentioned, the *cln3* deterministic circuit works only in the rDNA copy number amplification sense. This differs from other cases in which USCR interchange repeats between the two chromatids in other organisms or genome loci [12,31].

Finally, a deterministic circuit should have a feedback branch to shut down the mechanism, which generates genome variability once the optimal genome has been obtained to avoid runaway outcomes (Fig 3B). As far as we know, the yeast *cln3* evolution that we describe here is the only described case of an evolutionary deterministic circuit in a living being. The appearance of this kind of autoregulated circuits has obviously been driven by natural selection, but not to necessarily allow deterministic evolution. Such a circuit has most likely evolved because it allows the efficient regulation of physiological responses, such as the adaptation of the rDNA copy number to the yeast cell volume for optimal RNA pol I transcription and genome integrity [13–16]. The circuit can, however, be activated by unusual circumstances (e.g. artificial sudden increase in the cell volume of a *cln3* mutant) and drive a kind of evolution that has only been described to date in non living systems. Minisatellites are another potential candidate for a deterministic circuit. In this case, the change in copy number occurs by a different intra- or interchromatid recombination, which can lead to loss or gain of repeats [10]. In this case, amplification is skewed because repeated USCR biases the distribution of array lengths if long arrays tend to have greater absolute misalignment in crossovers than short arrays [10]. Thus once the mechanism is activated by the presence of several repeats, it should provoke deterministic evolution toward longer arrays until they become too costly in energy terms and it is removed by natural selection.

It is often assumed that living beings evolve by the selective pressure caused by the natural selection of the fittest. This Darwinian evolution is complemented in a variable proportion with the accumulation of neutral variations [40,41] that do not influence the phenotype and living beings’ capacity to compete with other individuals of the same species (Fig 3A). There should first be a more or less randomly produced source of variability for any kind of selection to occur between different genomes. Genetic variability can be caused by replication errors, external mutagenic agents, transposition or recombination mechanisms [34; Fig 3A]. Non biological systems without genetic information can also evolve when perturbed toward different states or compositions, but they follow a path guided by physico-chemical laws [36] in a predictable (deterministic) way (Fig 3A). This is the way for a totally different evolution from the Darwinian one, which has not been observed to date for living beings.

## Supporting information

S1 Fig Cellular automaton model accurately predicts the experimentally observed evolution of early *cln3*. A) Scheme of the algorithm used in Model A (Fig 2A). B) Number of generations and times required to obtain <98% of cells with 220 rDNA repeats and an average generation increase (Delta) between 0.3 and 2 copies. A Delta between 1.2 and 1.4 fits the experimental results (between 160 and 180 generations).
(PDF)

S2 Fig Cellular automaton model by assuming that *SIR2* repression linearly decreases. In this case, the cells with 125 copies divide 100% into a daughter with an amplified copy number by a given Delta factor and another cell with no amplification (125 copies). However, the cells with >125 copies have a linear increasing tendency (from 1% to 126 copies to 100% with 220 copies) to divide into two cells with no amplification. The table and the plot show the number of generations required for every possible integral Delta value. Note that only Delta >11 fits the number of experimentally observed generations.
(PDF)

S3 Fig Cellular automaton model by assuming that growth rate differences between cells have different rDNA copy numbers for models B. Scheme of the algorithm used for Models B (Fig 2D and 2E). The figure shows that the growth rate increases (generation time decrease, GTI -9 s). A model for a lowering growth rate would be similar, but with a GTI of +9 s.
(PDF)

S1 Table List of the yeast strains used in this work. (PDF)

S1 Appendix Algorithm used in Model A1. This appendix describes the pseudocode of Model A1. Both the implementation details and source codes for all the models can be downloaded from https://www.uv.es/varnau/modelo/MODEL_A.c.
(PDF)
