# Supplementary figures and images for: A feedback mechanism controls rDNA copy number evolution in yeast independently of natural selection
Source: PLoS One. 2022 Sep 1;17(9):e0272878. doi: 10.1371/journal.pone.0272878 (PMC9436098; doi:10.1371/journal.pone.0272878)

A) **Model A: constant growth rate**

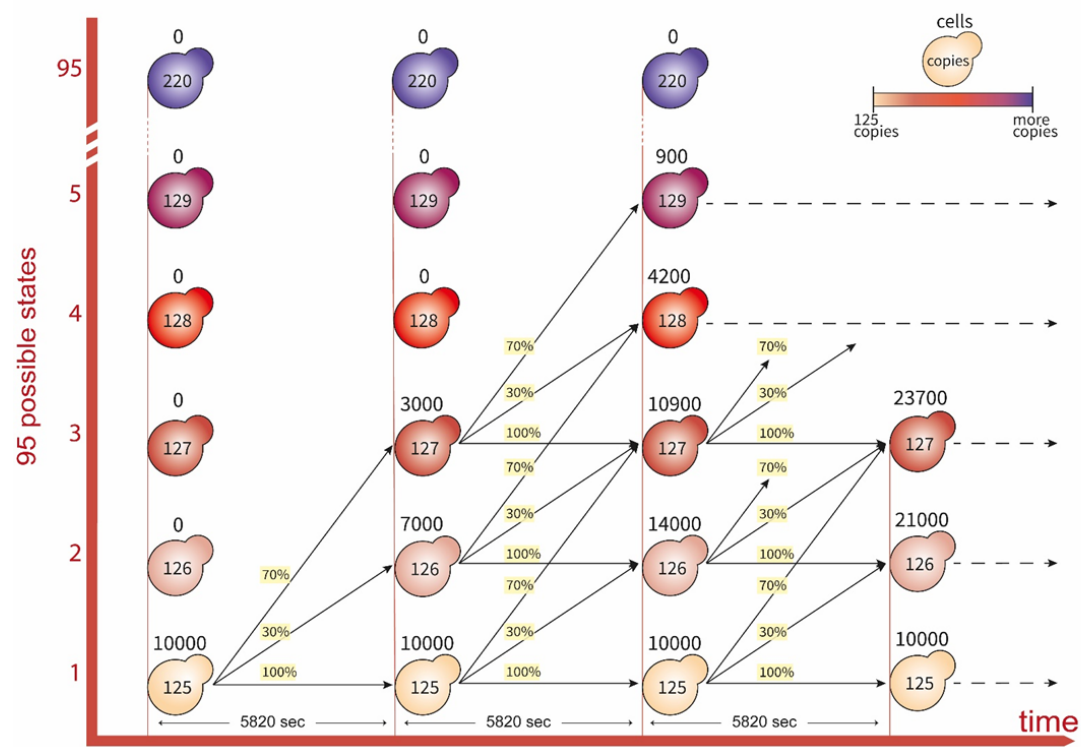

B) **MODEL A1**  
No reinoculation

| DELTA | Generations | Time (sec) |
|-------|-------------|------------|
| 0.3   | 763         | 4440660    |
| 0.4   | 569         | 3311580    |
| 0.5   | 453         | 2636460    |
| 0.6   | 375         | 2182500    |
| 0.7   | 320         | 1862400    |
| 0.8   | 278         | 1617960    |
| 0.9   | 246         | 1431720    |
| 1     | 220         | 1280400    |
| 1.1   | 203         | 1181460    |
| 1.2   | 189         | 1099980    |
| 1.3   | 176         | 1024320    |
| 1.4   | 164         | 954480     |
| 1.5   | 154         | 896280     |
| 1.6   | 145         | 843900     |
| 1.7   | 137         | 797340     |
| 1.8   | 130         | 756600     |
| 1.9   | 123         | 715860     |
| 2     | 118         | 686760     |

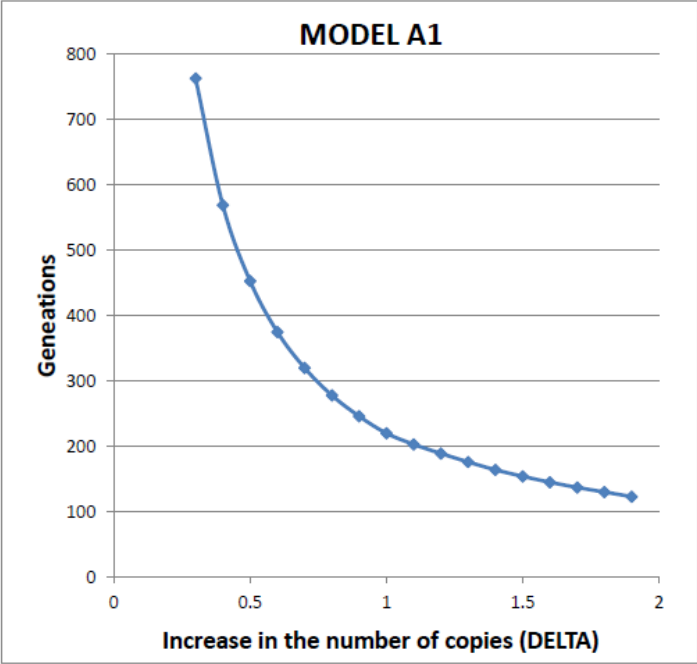

Figure S1

Supplement: S1 Fig — A) Scheme of the algorithm used in Model A (Fig 2A). B) Number of generations and times required to obtain <98% of cells with 220 rDNA repeats and an average generation increase (Delta) between 0.3 and 2 copies. A Delta between 1.2 and 1.4 fits the experimental results (between 160 and 180 generations). (PDF) [file pone.0272878.s001.pdf]

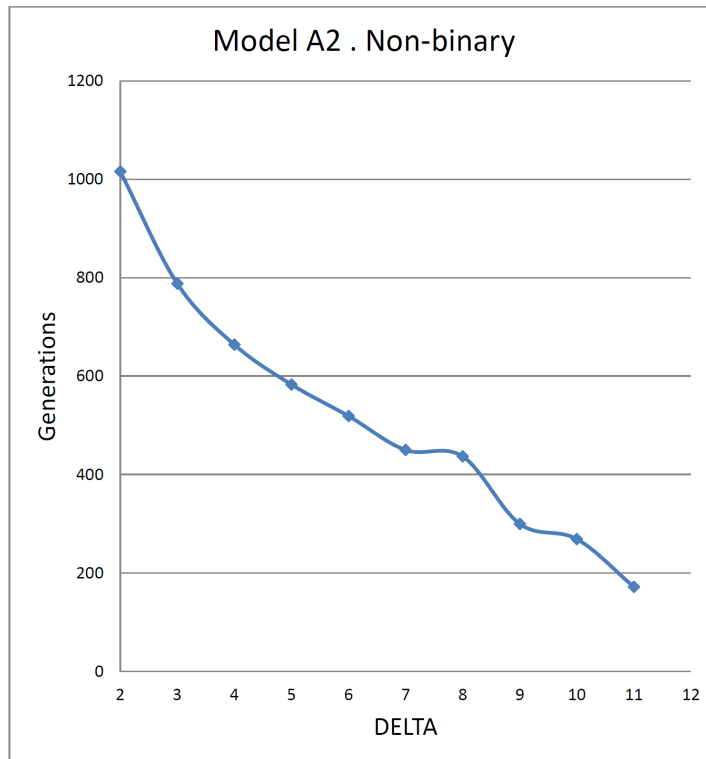

| Model A2. Non-binary |             |
|----------------------|-------------|
| DELTA                | Generations |
| 2                    | 1016        |
| 3                    | 788         |
| 4                    | 664         |
| 5                    | 583         |
| 6                    | 519         |
| 7                    | 450         |
| 8                    | 437         |
| 9                    | 300         |
| 10                   | 269         |
| 11                   | 172         |

Figure S2

Supplement: S2 Fig — In this case, the cells with 125 copies divide 100% into a daughter with an amplified copy number by a given Delta factor and another cell with no amplification (125 copies). However, the cells with >125 copies have a linear increasing tendency (from 1% to 126 copies to 100% with 220 copies) to divide into two cells with no amplification. The table and the plot show the number of generations required for every possible integral Delta value. Note that only Delta >11 fits the number of experimentally observed generations. (PDF) [file pone.0272878.s002.pdf]

## Model B: variable growth rate

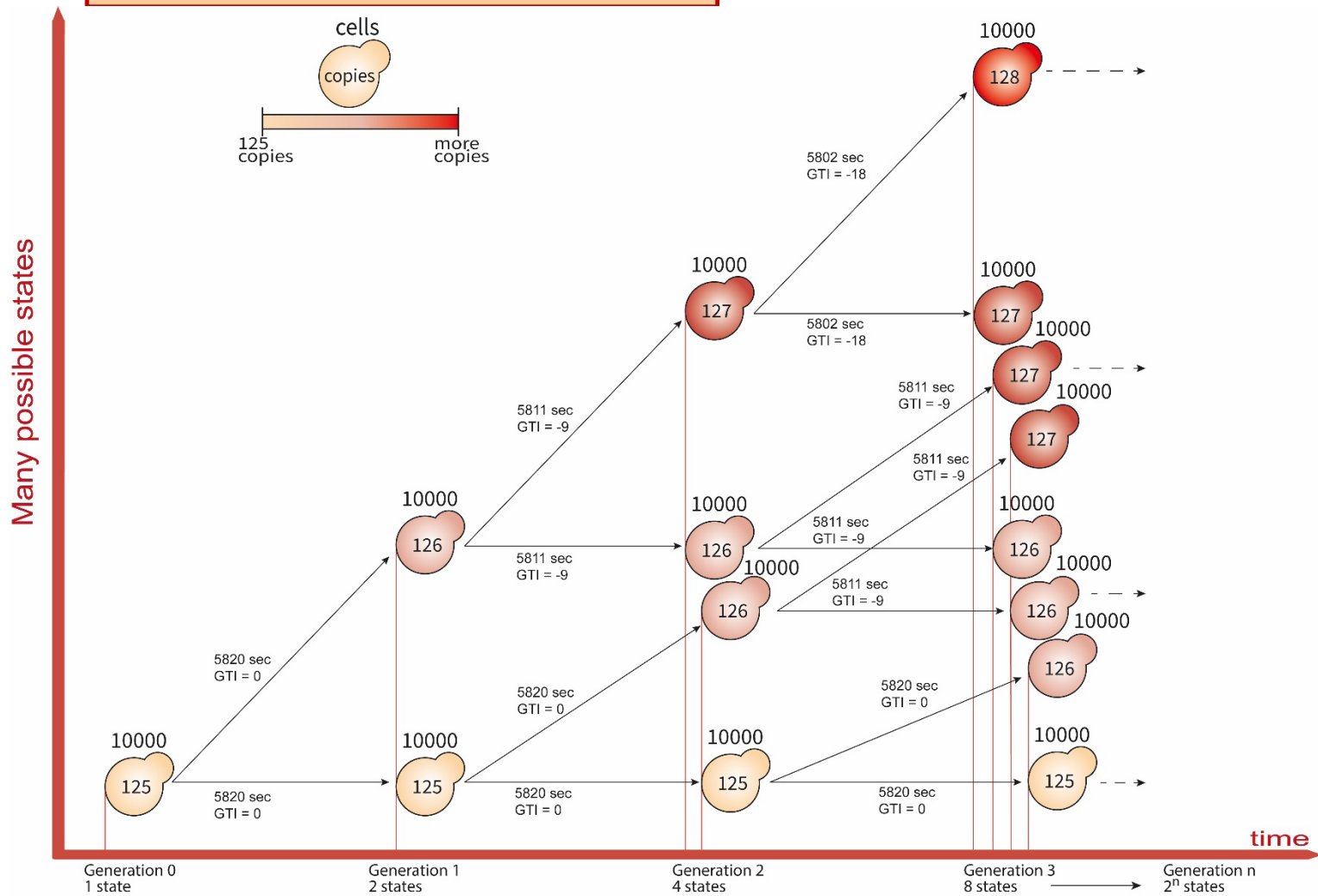

**Figure S3**

Supplement: S3 Fig — Scheme of the algorithm used for Models B (Fig 2D and 2E). The figure shows that the growth rate increases (generation time decrease, GTI -9 s). A model for a lowering growth rate would be similar, but with a GTI of +9 s. (PDF) [file pone.0272878.s003.pdf]
