# Supplementary material for: A feedback mechanism controls rDNA copy number evolution in yeast independently of natural selection
Source: PLoS One. 2022 Sep 1;17(9):e0272878. doi: 10.1371/journal.pone.0272878 (PMC9436098; doi:10.1371/journal.pone.0272878)
Supplement: S1 Table — (PDF) [file pone.0272878.s004.pdf]

Table S1. Yeast strains used in this study

Name	Genotype	Cell Volume (fL)	Source
**BY4741**	MATa *ura3Δ0 leu2Δ0 his3Δ1 met15Δ0*	50±4	Euroscarf
**BQS2006 (Euroscarf *cln3Δ*)**	MATa; *ura3Δ leu2Δ HIS3 MET15 lys2Δ cln3Δ::KanMX4*	83±10	Euroscarf
**BQS2078 (Early *cln3Δ*)**	MATa *ura3Δ0 leu2Δ0 his3Δ1 met15Δ0 cln3Δ::KanMX4 20 generations*	85±9	Ref. 16
**BQS2063 (Late *cln3Δ*)**	MATa *ura3Δ0 leu2Δ0 his3Δ1 met15Δ0 cln3Δ::KanMX4 200 generations*	85±10	Ref. 16
