# Supplementary material for: A feedback mechanism controls rDNA copy number evolution in yeast independently of natural selection
Source: PLoS One. 2022 Sep 1;17(9):e0272878. doi: 10.1371/journal.pone.0272878 (PMC9436098; doi:10.1371/journal.pone.0272878)
Supplement: S1 Appendix — This appendix describes the pseudocode of Model A1. Both the implementation details and source codes for all the models can be downloaded from https://www.uv.es/varnau/modelo/MODEL_A.c. (PDF) [file pone.0272878.s005.pdf]

APPENDIX

We describe here the algorithm used in model A1, without making use of re-inoculations, for a real value of DELTA.
Our cellular automata uses an array Estate[] of 96 states, from 95 to 225 state, and we use an auxiliary array Aux_Estate[] of same size.
The mod() function returns the integer part of a real number.

```
-------------------------
DEFINE  DELTA  1.3
DEFINE  MIN_COPIES   125
DEFINE  MAX_COPIES   220
DEFINE  INIT_CELLS 10000

Factor_1 = (Mod(DELTA)+1)-DELTA
Factor_2 = DELTA - (Mod(DELTA))

FOR (i=0 to (i <= MAX_COPIES))
    BEGIN
         Estate[i]= 0;  Aux_Estate[i]= 0;
    END;
Estate[MIN_COPIES] = INIT_CELLS;


REPEAT
BEGIN
      FOR (i=MIN_COPIES to (i <=MAX_COPIES))
      BEGIN
         Aux_Estate[i] = Aux_Estate[i] + Estate[i];
         Index_1 = i + Mod(DELTA);
         Index_2 = i + (Mod(DELTA) + 1);
         IF (Index_1 > MAX_COPIES) Index_1= MAX_COPIES;
         IF (Index_2 > MAX_COPIES) Index_2= MAX_COPIES;

         Aux_Estate[Index_1] = Aux_Estate[i] + Estate[i]*Factor_1;
         Aux_Estate[Index_2] = Aux_Estate[i] + Estate[i]*Factor_2;
      END

      FOR (i=MIN_COPIES to (i <=MAX_COPIES))
      BEGIN
             Estate[i] = Aux_Estate[i];
             Aux_Estate[i] = 0;
      END
END
WHILE ("unsaturated system");

---------------------------------------------------
```
